# Adaptive Control of Exoskeleton Robots for Periodic Assistive Behaviours Based on EMG Feedback Minimisation

**DOI:** 10.1371/journal.pone.0148942

**Published:** 2016-02-16

**Authors:** Luka Peternel, Tomoyuki Noda, Tadej Petrič, Aleš Ude, Jun Morimoto, Jan Babič

**Affiliations:** 1 Dept. of Automation, Biocybernetics and Robotics, Jožef Stefan Institute, Ljubljana, Slovenia; 2 Dept. of Brain Robot Interface, ATR Computational Neuroscience Labs, Kyoto, Japan; Shanghai Jiao Tong University, CHINA

## Abstract

In this paper we propose an exoskeleton control method for adaptive learning of assistive joint torque profiles in periodic tasks. We use human muscle activity as feedback to adapt the assistive joint torque behaviour in a way that the muscle activity is minimised. The user can then relax while the exoskeleton takes over the task execution. If the task is altered and the existing assistive behaviour becomes inadequate, the exoskeleton gradually adapts to the new task execution so that the increased muscle activity caused by the new desired task can be reduced. The advantage of the proposed method is that it does not require biomechanical or dynamical models. Our proposed learning system uses Dynamical Movement Primitives (DMPs) as a trajectory generator and parameters of DMPs are modulated using Locally Weighted Regression. Then, the learning system is combined with adaptive oscillators that determine the phase and frequency of motion according to measured Electromyography (EMG) signals. We tested the method with real robot experiments where subjects wearing an elbow exoskeleton had to move an object of an unknown mass according to a predefined reference motion. We further evaluated the proposed approach on a whole-arm exoskeleton to show that it is able to adaptively derive assistive torques even for multiple-joint motion.

## Introduction

The enormous potential of robotic mechanisms has been partially realised in industry where they perform various repetitive tasks. While being very useful, these robots are designed to mostly work separately from the humans. In future we expect to move the robots from the structured industrial environments into the human daily lives. Two of the main research fields pursuing this goal are service robotics and rehabilitation robotics where the robots are expected to work with the humans and adapt to their behaviour. A promising direction in these areas are exoskeleton assisting devices that have been developed in the last decades [1]. These robotic mechanisms are designed to enclose the human limbs and augment their existing capabilities or substitute the impaired ones by adding additional power to the joints.

The development of powerful, efficient, adaptable, lightweight and low-cost mechanisms [1–8] is imperative in achieving applicable exoskeletons and fulfilling the goal of integration of robots into our daily lives. On the other hand, equally important are the control methods that provide the necessary interface between the human user and robotic mechanism. The control framework should be able to predict the human intentions and apply them to the mechanism at the correct time to achieve the desired human-robot cooperation and co-adaptation.

Some researchers proposed control methods where the human intention was detected through the use of human-robot interaction dynamics. In one such example, Pratt et al. [9] built a knee exoskeleton called *RoboKnee* that was powered by series elastic actuators. They measured the change of the spring element length in-between the load and the motor to determine the direction and magnitude of the motor force. Kong and Jeon [10] controlled the assistive behaviour of lower-limb exoskeleton based on the pressure measured between the human limb and exoskeleton frame. In another instance, *Berkeley Lower Extremity Exoskeleton* (BLEEX) [3] was controlled by a hybrid approach [11] to achieve assistance in different phases of the gait cycle. This method used inverse dynamics model in combination with positional controller in stance phase, and positive-feedback based sensitivity amplification controller in the swing phase. Tsagarakis and Caldwell [5] proposed an impedance control scheme for an upper-limb rehabilitation exoskeleton. Ronsse et al. [12] and Gams et al. [13] used dynamical models to support elbow and knee movements, respectively. One of the advantages of the above-mentioned control methods is that they can operate based on the signals measured on the exoskeleton.

Measuring interaction dynamics and using inverse dynamics models can be an effective exoskeleton control approach to assist the able-bodied human subjects. However, the nature of such methods may not be suitable in certain cases, as the user must be able to produce a certain torque in the joints to initiate the motion before the exoskeleton can offer support [14]. If the users are unable to produce sufficient torques in their joints, the robot may not be successfully controlled. This disadvantage can be avoided by measuring human intention from biological signals that are sent from the central nervous system (CNS) to the motor units. A non-invasive surface electromyography (EMG) provides a convenient way to extract the muscle activation commands by detecting the electrical potentials on the skin above the muscles. EMG signals have been successfully applied in exoskeleton control in the past [2, 14–20]. The crucial advantage of EMG-based methods is that even if the subject is unable to produce sufficient joint torques, the intention of the human user can still be read and consequently the exoskeleton can be controlled. This is under the assumption that EMG signals with sufficient information can be extracted. Due to the advantages of biological signals, we selected EMG-based biofeedback in our method.

Several EMG-based exoskeleton control methods are using biomechanical models to estimate the output joint torque based on the muscle activity measured by EMG [14, 15, 18, 20]. As an alternative to biomechanical models, some researchers proposed the use of artificial neural networks to learn this complex relation [16, 21] or statistical learning algorithms for classification of different action modes or motion patterns from the measured EMG signals [22, 23]. On the other hand, some studies [2, 17, 19] showed that a simple proportional mapping between EMG-based muscle activity and control output can achieve satisfactory control results. The concept of these methods is to amplify the current human behaviour, which requires a continuous muscular effort even if the task is repetitive. In case of repetitive tasks, where the joint motion is periodic, it is reasonable to offload the human user of some of the effort by learning and repeating the desired motion.

If the joint motion is repetitive, it is convenient to encode it with trajectories and then reproduce them. Dynamical Movement Primitives (DMP) is one of the methods for parametric representation of trajectories [24, 25]. Frequently, the phase and frequency of trajectories are extracted and controlled by Adaptive Frequency Oscillators [26–29]. The adaptive oscillators have been also used in exoskeleton control. For example, Ronsse et al. [30] used adaptive oscillators to adapt the frequency of the positional trajectories that were learnt according to the subject’s motion. The method estimated the assistive joint torque based on the difference between the current position and predicted phase-shifted future position.

In other instances, researchers used adaptive oscillators to directly control the frequency of predetermined feed-forward torque behaviour. Ronsse et al. [12] and Gams et al. [13] used adaptive oscillators to control the phase of exoskeleton assistive torque estimated by inverse dynamics models. Similarly, Aguirre-Ollinger [31] used adaptive oscillators to control the phase of periodic hip joint torque profile that was previously estimated from the measured human muscle activity. Aforementioned examples of periodic exoskeleton assistance used the adaptive oscillators to drive the phase and reproduce the model-determined or pre-estimated joint torque behaviour at a certain frequency. During the execution of the task, the robot behaviour adapts to the human pace by modulation of frequency of given periodic torque profile. However, if the human changes the behaviour, for which model or pre-estimated torque trajectories do not exist, then the new models/trajectories need to be obtained. This requires switching from human-assistance operation mode to a mode where the new models/trajectories can be estimated.

Contrary to [12, 13, 31], in this paper we propose an approach that is not based on just repeating the pre-modelled or pre-estimated torque behaviour. Our control method dynamically adapts the shape of the robot joint torque trajectories in accordance to the current human behaviour. Rather than using muscle activity to estimate the torque profile as in [31], we use muscle activity as an information in which direction should the controller adapt the learnt torque trajectories to minimise that muscle activity in the corresponding phase of periodic cycle. The key advantage of the proposed method compared to [12, 13, 31] is that it does not require to switch from assisting to learning mode if a new exoskeleton behaviour is required. The new exoskeleton feed-forward behaviours are gradually formed during the operation mode.

The proposed method is similar to positive-feedback controllers in a sense that both use positive feedback signal. However, a crucial conceptual difference is in how the feedback is utilised. In positive-feedback controllers the feedback is used in a direct fashion to control the motion (measured torque amplification, EMG-to-torque mapping, etc.). On the other hand, in our method it is the learnt feed-forward behaviour that controls the motion, while the feedback is (only) used to gradually update the feed-forward behaviour. Therefore, the proposed method primarily a feed-forward control scheme, while positive-feedback controllers are primarily a feedback control scheme. As the proposed method is primarily a feed-forward control scheme it inherits some of the robustness of the feed-forward control and is not as easily prone to feedback-related instabilities as positive-feedback controllers.

An advantage of the proposed method is that it does not simply amplify the effort (i.e. torque, motion) produced by the user. Instead, it aims to take over the effort required for the task execution and therefore allows the exoskeleton to move and execute the task even when the human muscles are relaxed. Compared to positive-feedback control framework, where continuous muscular effort is required to control the exoskeleton, our method requires less muscular effort from the user in case of sustained periodic tasks. In addition, compared to methods that require inverse dynamics models [3, 5, 7, 11–13] or EMG-to-torque estimation models [14, 15, 18, 20], our approach is model-free and does not require time-consuming and error-prone modelling and calibration processes. The adaptation of the joint torque profile based on the minimisation of human muscle activity feedback bypasses the need of human, robot and task dynamics models. The method provides the means to acquire the desired trajectories even when dynamical models are not available.

We utilise periodic DMPs to encode torque-control trajectories and couple them with adaptive oscillators to control their phase and frequency. The existing DMP learning was modified in order to achieve a mode of operation where the trajectory gradually adapts based on the human biofeedback. The muscle activity feedback provides information on how the assistive exoskeleton trajectory should be updated to minimise that feedback. This allows the human user to adapt the periodic trajectories of motion during the task execution. The proposed learning approach aims at making the exoskeleton adapt to humans and assist them in a desired repetitive task. Compared to the positive-feedback framework methods, in our approach the user does not need to constantly produce high effort. Instead, the robot takes over the execution of the periodic task when no changes are required. The user only needs to make an effort when the learnt behaviour is to be modified. The proposed method provides a human user with the ability to update the trajectory in real-time through the use of biofeedback when the circumstances call for modifications. Additional advantage is that the learnt behaviour can be stored for their immediate reproduction in future.

The main goal of this paper is to propose and validate the exoskeleton control method. We see a potential applicability of this approach in some power-assist and rehabilitation scenarios. For example, suitable tasks in case of power-assist may include: walking, where in case of covering a longer distances the exoskeleton behaviour is periodic; repetitive household tasks such as wiping a table; and industrial tasks such as picking heavy objects from a conveyor belt. Some rehabilitation exercises include repetitive training [5–7, 32, 33] and could be performed using the method proposed in this paper. To validate the method we designed several experiments with a general task of moving an object with unknown mass. This task can relate to many daily human arm tasks or rehabilitation exercises. The experiments included qualitative analysis using a trained subject and a multi-subject analysis.

## Hardware Setup

The experiments were performed on two different mechanical systems. First we tested a general feasibility of the proposed approach by performing experiments on an elbow exoskeleton in forearm motion assistance. To show that we can deal with more complex tasks, we then expanded the setup to a whole-arm exoskeleton using two active degrees-of-freedom (DoF). Both systems operated at a frequency of 250 Hz. In the end we performed a multi-subject analysis on the elbow exoskeleton to examine the applicability of the method on a wider range of users.

**Fig 1:**
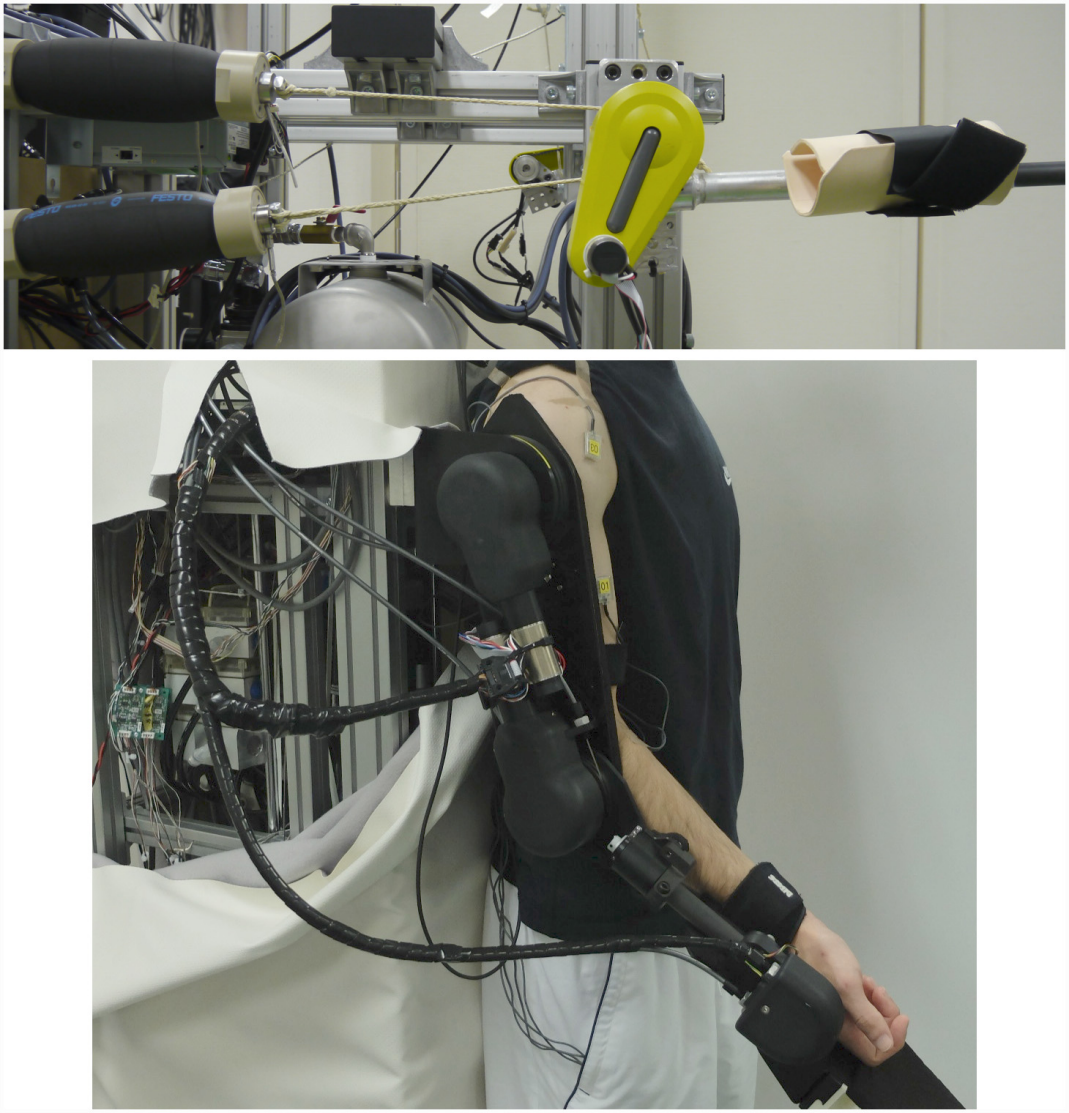
Exoskeleton systems used in the experiments, developed at BRI ATR. The top photo shows the elbow exoskeleton [34] and the bottom photo shows the whole-arm exoskeleton [35].

The elbow exoskeleton [34] (see Fig 1, top) that we used in the initial series of experiments was developed at BRI ATR and was mainly designed for rehabilitation. It was driven by two antagonistically working Festo MAS-40 pneumatic artificial muscles (PAM), which were connected to a rotational joint by string tendons. For the given weight lifting tasks only the upper PAM was employed, considering the acceleration in downward direction beyond the gravity was not required/desired. A load cell was fixed in-between the string and the muscle to measure the torque induced into the joint. The radius of the joint was 0.024 m. The human forearm was enclosed by a plastic base, which was previously shaped to fit the subject’s forearm. The length of the exoskeleton main forearm segment was 0.3 m. The tip of the segment allowed mounting of additional extension parts where the weights were fixed.

The whole-arm exoskeleton [35] (see Fig 1, bottom), developed at BRI ATR, had 3 active joints: shoulder, elbow and wrist flexors/extensors. Each joint is actuated by two antagonistically working PAMs located separately from the arm. The actuation was done through bowden cables connected to the joints. The length of the arm segment was 0.263 m, the length of forearm segment was 0.252 m and the length of artificial hand was 0.2 m. During the experimental evaluation we considered an end-effector at the human palm, approximately 0.055 m from the robot wrist joint. The joint radius of shoulder joint was 0.05 m and of elbow joint 0.038 m. The human arm was fixed to the robotic arm segments by straps.

We controlled the torque produced in the joints by the pressure inside PAMs. Previously some researchers used complex feed-forward models to define the relation between air pressure and resulting muscle force [36, 37]. The same internal pressure produces different forces at different muscle lengths due to the change in volume. The models must predict the necessary control pressure inside the muscle to produce the desired output force at different muscle lengths. Modelling can be difficult due to the highly non-linear relation between these control variables and influence of other mechanical effects. Deviations between the real and predicted behaviour may cause errors and undesired behaviour in torque-control mode. A measured force feedback may be implemented to compensate the errors in the feed-forward model [5, 38–41]. However, additional force sensors may significantly increase the cost and applicability of exoskeletons. In addition, the feedback loop may be prone to induce unwanted delays and oscillations if the measured force is noisy.

By using the proposed control method, it is possible to bypass PAM pressure-to-torque models by directly learning the pressure trajectories instead of torque trajectories. The adaptable nature of the proposed method shapes a pressure trajectory that produces the necessary torque to minimises the muscle activity feedback signal. If the produced robot joint torque deviates from the torque required to compensate the desired human joint behaviour (i.e. human still has to produce some effort), the muscle activity feedback should update PAM pressure to appropriately adapt the torque. To demonstrate this advantage we decided to directly learn the pressure trajectories.

## Control Method

The basic idea/concept of the proposed exoskeleton control approach is to minimise the human muscle activity feedback by learning the appropriate assistive torque behaviour in the joint. In this sense, the control approach is mainly feed-forward based, while the feedback from the human user is used to adapt the learnt feed-forward control policy. We can describe the single joint equilibrium dynamics as

$$\begin{array}{c} \tau_{e}\left(\phi \right)+\tau_{m}\left(\phi \right)=\tau_{l}\left(\phi \right), \end{array}$$ (1)

where *τ*_*e*_(*ϕ*) is a phase-dependant learnt feed-forward torque produced by the exoskeleton, *τ*_*m*_(*ϕ*) is the torque produced by the human muscle and *τ*_*l*_(*ϕ*) is the torque of the load and other dynamics at a given phase *ϕ*.

The current torque *τ*_*m*_ produced by the muscle is a function of muscle properties *μ* and muscle activation level *A* [14]

$$\begin{array}{c} \tau_{m}=f\left(\mu ,A\right). \end{array}$$ (2)

A simplified single-muscle expression that describes the proposed control approach is given as

$$\begin{array}{c} \Delta \tau_{e}^{k}\left(\phi \right)=G\left(A^{k-1}\left(\phi \right)\right), \end{array}$$ (3)

where $\Delta \tau_{e}^{k}$ is the change of the learnt exoskeleton torque $\tau_{e}^{k}$ at a given phase *ϕ* in the current cycle/iteration *k* based on the muscle activity *A*^*k*−1^ in the previous cycle/iteration. Function *G* maps the muscle activation level to the change of learnt torque.

According to the properly selected function *G*, this system will minimise the muscle activity as measured by EMG. If the human muscle has to produce the torque *τ*_*m*_ to compensate the load *τ*_*l*_, the activation level *A* is non-zero and Δ*τ*_*e*_ will be changing the exoskeleton learnt torque τ_*e*_ until it compensates *τ*_*m*_. Assuming that τ_*l*_ is fully compensated by the exoskeleton, then the muscle activation level *A* should be ideally zero. Since muscle activity related EMG reading is non-zero even when muscles are relaxed, various bias or threshold techniques should be applied to ensure *A* is zero when the muscle is relaxed. When *A* falls to zero, Δ*τ*_*e*_ should become zero and the leant behaviour will endure until some non-zero *A* is produced either by the change of load/conditions or by a voluntary intention of the human. For example, if the frequency of the already learnt motion is increased then the currently learnt torque throughout the phase will not match the new dynamical behaviour and the human muscles will have to compensate until the torque trajectories are adapted.

Note that Eqs (1)–(3) describe a simplified single-muscle case for purpose of illustrating the concept. Therefore, in this example the learnt torque can only increase since muscle activity cannot be negative. For bi-directional change of the learnt torque, a signal composed of antagonistic pair of muscle activations is required. This is explained in the next section.

Assuming that the human motion and behaviour are stable, we can analyse the stability of simplified model of our approach using Eqs (1)–(3). At the beginning, the human muscle has to compensate the entire torque of load *τ*_*l*_ while the learnt exoskeleton torque *τ*_*e*_ is zero. Theoretically, our approach described by Eq (3) will be stable if the mapping function *G* updates the exoskeleton torque *τ*_*e*_ by less or equal the torque of load *τ*_*l*_. If the update is equal to the torque of the load *τ*_*l*_ then the exoskeleton torque *τ*_*e*_ takes the entire load, the muscle activity *A* is zero and the updating stops (system is settled down). If the exoskeleton torque *τ*_*e*_ is larger than the torque of the load *τ*_*l*_ then the system could potentially become unstable. However, since human is coupled with the exoskeleton, his/her reaction will trigger new torque updates, to perform desired task and keep the system stable. To theoretically determine the exact value of *G* limit one would require models of the system. Since in our method we do not have/use models, we have to empirically determine the appropriate value range of *G*. This should be done on a conservative basis so that the adaptation is gradual and therefore the system operates well within the stability limits.

The stability of the system can be also affected by the execution frequency (i.e. change of phase *ϕ*). Several factors should be considered when analysing the upper frequency bound. Human neural feedback delay [42] and dexterity of the individual user limit the human response time. The delay in pneumatic actuators limits the response time of the exoskeleton [41]. Feasible execution frequency upper bound is also affected by the dynamical aspects, such as task (especially motion range), load, exoskeleton limb, human limb and other conditions. High execution frequency may result in very high inertial forces that cannot be physically produced or sustained by the robot or human. Without the exact models of the system under the given task/conditions, the exact frequency bound cannot be obtained. Since in our method we do not have/use the models, we have to determine this bound empirically on a conservative basis.

**Fig 2:**
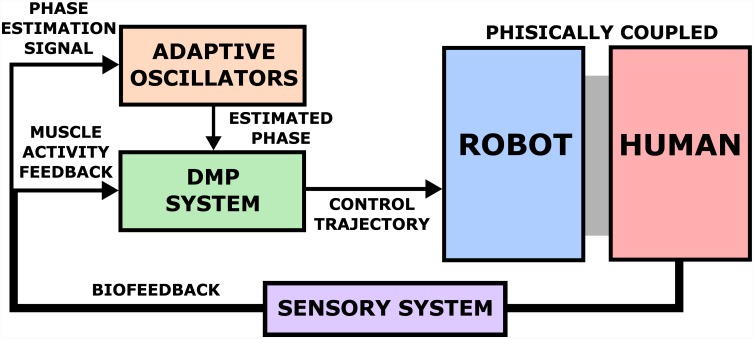
Schematic presentation of proposed adaptive exoskeleton control system. The human is included into the robot control loop through EMG-based biofeedback and DMP learning system. DMP system was made to minimise the EMG-based biofeedback by generating appropriate periodic feed-forward exoskeleton control trajectories. The adaptive oscillators are used to extract the phase and frequency information from human biofeedback and modulate the phase and frequency of the learnt feed-forward trajectories.

The practical implementation of the proposed exoskeleton control system for periodic tasks is shown in Fig 2. It is based on learning and updating the trajectories of motion in real-time. Pressure trajectories are shaped through the human muscle activity feedback signals and control exoskeleton joint torques. To encode pressure trajectories we use periodic Dynamical Movement Primitives [25] which offer parametric representation. Among the key advantages of DMPs are that they can represent both point-to-point and rhythmic movements, are learnable, are not explicitly dependant on time, are robust to perturbations, can include coupling terms to realise closed-loop reactive behaviours, and can easily be modulated on-line to change various movement characteristics [24]. Some of these properties can be achieved by *spline* methods that use interpolation techniques to connect several stored via points. However, they are time-based, susceptible to perturbations and their modulation is computationally more costly compared to modulation of DMP where only a few parameters need to be updated [24]. Another reason for choosing DMPs was the existence of extensive studies on their coupling with Adaptive Frequency Oscillators [27–29].

Shaping and reshaping of the desired trajectories was done by Locally Weighted Regression [43]. We employed Adaptive Frequency Oscillators [27, 28] to estimate periodic parameters of the output trajectories. The adaptive oscillators allow the human user to control the phase and frequency of the trajectories through the selected biofeedback signal.

### Biofeedback-Based Control Signal

The biofeedback we used to adapt the pressure trajectories was obtained through surface EMG. We attached EMG electrodes to the skin over the relevant muscles to extract the information about the individual muscle activity in the antagonist pairs. Each measured EMG signal was rectified and filtered by a 2nd-order Butterworth low-pass filter. The processed signals were then normalised using a classical isometric Maximal Voluntary Contraction (MVC) method [44, 45]. The amplitude normalisation minimises the effects of various variability factors, such as electrode configuration, and enables comparison between EMG signals [45]. Muscle activation levels for each muscle in antagonistic pair were obtained by

$$\begin{array}{cc} 0\leq A_{1}\left(t\right) & =\frac{EMG_{1}\left(t\right)-B_{1}}{MVC_{1}-B_{1}}\leq 1, \end{array}$$ (4)

$$\begin{array}{cc} 0\leq A_{2}\left(t\right) & =\frac{EMG_{2}\left(t\right)-B_{2}}{MVC_{2}-B_{2}}\leq 1, \end{array}$$ (5)

where *A*_1_(*t*) and *A*_2_(*t*) are a pair of muscle activation levels, while *EMG*_1_(*t*) and *EMG*_2_(*t*) are a pair of processed EMG signals extracted from agonist and antagonist muscle, respectively. *MVC* is the EMG signal produced by the maximal voluntary contraction of the muscle and *B* is the EMG signal measured when the muscle was relaxed. Bias *B* makes the muscle activation feedback equal to zero when muscle is in a relaxed state, which in turn stops the trajectory adaptation when exoskeleton takes over the task execution. Parameters *MVC* and *B* were obtained by averaging the measured EMG signal over a period of 3 seconds [44].

We combined the two antagonistic pairs/sets of muscle activity signals into a single feedback signal that was used to shape the control trajectory for the corresponding exoskeleton joint. Activation level of each muscle in the antagonistic pair defines the update direction for the trajectory of the corresponding joint. The adaptation feedback signal is defined as

$$U(t)=G_{u}\left(A_{1}\left(t\right)-A_{2}\left(t\right)\right),$$ (6)

where *G*_*u*_ is a scaling factor (related to function *G* from Eq (3)) that determines the intensity of the DMP trajectory update.

Feedback signal *U*(*t*) becomes the update value in the proposed DMP learning system. The goal is to adapt the joint torque trajectory and therefore gradually minimise the respective joint muscle activity required to perform the desired task. If the muscle activation level is high at a given phase of the trajectory, the feedback *U*(*t*) will adapt and reshape the trajectory in that region in a way that the produced torque in the robot joint will assist the human muscle in the desired task. The direction of the change depends on the combined muscle activity feedback signal acquired from Eq (6). If the agonist muscle has a predominant activation *A*_1_(*t*) > *A*_2_(*t*), the feedback *U*(*t*) will be positive and trajectory will update in a positive direction at that phase. In the opposite case, i.e. when the antagonist muscle has a predominant activation *A*_2_(*t*) > *A*_1_(*t*), the negative change of the trajectory will occur.

In this case, we selected a constant gain *G*_*u*_ as the mapping function *G* between muscle activation *A* and feedback signal *U* used for adapting the trajectory. The mapping could follow some other function *G*. For example, additional components could be added to damp the potential abrupt changes of *U*. However, the recursive regression update formula (see Eq (15) in the next section) and the DMP system dynamics inherently result in smooth trajectories and act similarity to a low-pass filter, therefore additional components were not necessary.

For the purpose of validation of the proposed control method, we used MVC normalisation based muscle activation model (descried by Eqs (4) and (5)). We relied on the human subject to adapt and compensate/minimise any practical deviations from the model. Since the human is inside the robot control loop, he/she can utilise the sensorimotor learning ability to adapt to controlling the robot under the given conditions [29, 46]. The proposed exoskeleton control approach actively adapts itself to the human behaviour through the use of human biofeedback. In return, the human sensory information (visual, tactile, proprioceptive) provides the feedback about the impact of human control signals on the current robot behaviour, which allows the subject to learn and correct the appropriate control policy within the given setup. However, more complex models of human muscle activation can be employed to cope with limb configuration related variations [47]. In addition, various techniques can be applied to further reduce the effects of other common surface EMG problems, such as baseline drift, noise and movement artefacts [48].

### Trajectory Encoding and Updating

Dynamical Motion Primitives (DMPs) [25] are based on a second order differential equation system described as

$$\begin{array}{c} \dot{z}=\Omega \left(\alpha \left(\beta \left(-y\right)-z\right)+f\right), \end{array}$$ (7)

$$\begin{array}{c} \dot{y}=\Omega z, \end{array}$$ (8)

where *y* is the quantity we wish to encode with the DMP, Ω is the frequency, *f* is the shape function, which appropriately modulates the second order differential equation, and *α* and *β* are positive constants. In our case, the trajectory *y* is the desired pressure control trajectory *p*. We selected parameters *α* = 8 and *β* = 2 according to [27]. The main difference between periodic DMP and point-to-point DMPs is that the time constant related to trajectory duration is replaced by the frequency of trajectory execution (refer to [24, 25] for details). In addition, the periodic DMP must ensure that the initial point (*ϕ* = 0) and the final point (*ϕ* = 2*π*) coincide in order to achieve smooth transition during the repetitions. Shape *f* is defined with Gaussian kernels according to the following equation

$$f\left(\phi \right)=\frac{{\sum_{i=1}^{N}\psi_{i}\left(\phi \right)w_{i}}_{}^{}}{{\sum_{i=1}^{N}\psi_{i}\left(\phi \right)}_{}^{}},$$ (9)

Gaussian kernel *ψ*_*i*_(*ϕ*) is phase dependant and defined as

$$\begin{array}{c} \psi_{i}\left(\phi \right)=e^{h\left(\cos\left(\phi -c_{i}\right)-1\right)}, \end{array}$$ (10)

where parameter *h* is the width of the Gaussian kernel, *c*_*i*_ are the uniformly distributed centres of the base functions within the phase range (i.e. between 0 and 2*π*) and *N* is the number of weights. The selection of the number of weights should be based on the desired resolution of the trajectory. In our experiments we used 25 weights. Refer to [24, 25] for details.

We applied Locally Weighted Regression [43] to learn the trajectory. In a standard periodic DMP setting [25, 27], the desired shape *f*_*d*_ is approximated by solving

$$\begin{array}{c} f_{d}=\frac{{\ddot{y}}_{d}}{\Omega^{2}}-\alpha \left(\beta \left(-y_{d}\right)-\frac{{\dot{y}}_{d}}{\Omega }\right), \end{array}$$ (11)

where *y*_*d*_ is some demonstrated input trajectory that needs to be encoded. Weights *w*_*i*_ of Gaussian kernel functions *ψ*_*i*_ can be updated using the recursive least squares method [43] with forgetting factor λ based on the error between the desired trajectory shape and currently learnt shape [27]

$$\begin{array}{ccc} w_{i}\left(t+1\right) & = & w_{i}\left(t\right)+\Psi_{i}P_{i}\left(t+1\right)re_{r}\left(t\right), \end{array}$$ (12)

$$\begin{array}{c} e_{r}\left(t\right)=f_{d}\left(t\right)-w_{i}\left(t\right)r, \end{array}$$ (13)

$$\begin{array}{c} P_{i}\left(t+1\right)=\frac{1}{\lambda }\left(P_{i}\left(t\right)-\frac{P_{i}{\left(t\right)}^{2}r^{2}}{\frac{\lambda }{\Psi_{i}}+P_{i}\left(t\right)r^{2}}\right), \end{array}$$ (14)

where the initial state of the parameters are *w*_*i*_(0) = 0 and *P*_*i*_(0) = 1 for *i* = 1, 2, …, *N*. The forgetting factor was set to λ = 0.9995. Refer to [43] for details on parameter setting. Parameter r in the DMP system is used to modulate the amplitude of the periodic signal [25]. In our case we set it to *r* = 1 and do not change it afterwards.

The standard DMP learning approach as proposed by Gams et al. [27] approximates the shape *f*_*d*_(*t*) of the input trajectory *y*_*d*_ by changing the weights of Gaussian kernel functions. The updating of the weights is performed in such a way that the difference between the reference trajectory and the DMP is reduced at every control step. Instead of providing a reference trajectory, Petrič et al. [49] defined the desired direction of trajectory modification *e*_*r*_(*t*) through pointing gestures, enabling the user to interactively alter existing DMPs. Here we modified this approach so that instead of pointing gestures, the muscle activity feedback signal provides information about the direction of joint torque change. With this we made a practical implemented our proposed exoskeleton control approach based on minimisation of human muscle activity feedback through trajectory adaptation. The human was included into the robot control loop by replacing the error calculation in Eq (13) with human muscle activity feedback *U*(*t*) as defined by Eq (6)

$$\begin{array}{ccc} w_{i}\left(t+1\right) & = & w_{i}\left(t\right)+\Psi_{i}P_{i}\left(t+1\right)r\left(t\right)U\left(t\right), \end{array}$$ (15)

Eqs (7)–(10) and (14) remain as before, while Eqs (11)–(13) are not used. Instead, Eq (15) is used to modulate the weights in Eq (9). Muscle activity *U*(*t*) closes the loop and now acts as an adaptation factor for changing the weights of Gaussian kernels that define the shape of the trajectory. A positive *U*(*t*) increases, while a negative *U*(*t*) decreases the values of weights at a given section of periodic trajectory. If the shape of assistive trajectory does not provide enough assistive power, the human has to exert muscle activity to produce the rest of the power required to achieve the desired task. In turn, muscle activity feedback then increases the magnitude of assistive trajectory until *U*(*t*) is minimised.

### Adaptive State Estimation

The joint torque-control trajectories were then coupled with adaptive frequency oscillator. The oscillator extracted the periodic parameters of the task (frequency, phase) from the task-related biofeedback signal and applied them to the trajectory learnt by the DMP system. The adaptive oscillator calculates phase *ϕ* and frequency Ω as [27, 28]

$$\begin{array}{cc} \dot{\phi } & =\Omega -K\cdot e\cdot \sin(\phi ), \end{array}$$ (16)

$$\begin{array}{cc} \dot{\Omega } & =-K\cdot e\cdot \sin(\phi ), \end{array}$$ (17)

where parameter *K* is a positive-value coupling constant and *e* is a variable that contains an input signal used for estimation. In our case, variable *e* is given as a difference between biofeedback signal *U* and an internal estimation of that signal $\hat{U}$

$$e=U-\hat{U},$$ (18)

where *U* is obtained by Eq (6) and is the input of adaptive oscillator from which the phase and frequency are extracted. Estimation $\hat{U}$ is performed by the use of Fourier series [28]

$$\begin{array}{cc} \hat{U} & =\sum_{c=0}^{{}^{M}}\left(\alpha_{c}\cos\left(c\phi \right)+\beta_{c}\sin\left(c\phi \right)\right), \end{array}$$ (19)

where *M* is the size of the Fourier series. Fourier series parameters are learnt in the following manner

$${\dot{\alpha }}_{c}=\eta \cos(c\phi )\cdot e,$$ (20)

$${\dot{\beta }}_{c}=\eta \sin(c\phi )\cdot e,$$ (21)

where parameter *η* is a learning constant. We selected *K* = 10, *M* = 10 and *η* = 2. Refer to [28] for details. The estimated phase *ϕ* and frequency Ω were used to control the currently learnt DMPs (in Eqs (7)–(10)). Alternatively, the phase and frequency could be controlled externally by some other feedback modality.

Previously some researchers that utilised the adaptive oscillators in inverse dynamics based exoskeleton control used positional signal (joint angle) as an estimation of the task phase and frequency [12, 13]. In case of exoskeleton, the human limb and robotic mechanism are physically coupled. If the learnt assistive trajectory induces a torque into the joint, the human subject has to produce an opposing torque to change the joint angle. In [12, 13] the exoskeleton compensated only a portion of the task load and frequency estimation through the joint angle was feasible. In our case however, the exoskeleton fully takes over the task load and the assistive torques are large. In such case the frequency control can be difficult. In addition, fighting large assistive torques with the aim to control the frequency produces muscle activity that may change the shape of the trajectory in undesirable way. In case of [12, 13] this was not the problem because muscle activity feedback was not used. We performed several preliminary experiments with joint angle signal for frequency extraction. The subjects experienced difficulties in altering the existing trajectories. Based on this and the above-mentioned specifics, we decided to use muscle activity feedback *U*(*t*) itself as a frequency extraction signal.

## Overview of Experiments

In the experiments the task of the human was moving an object of an unknown mass from one position to another position at a certain frequency, while the task of the exoskeleton was adapt to the human and offload the muscular effort. In the first set of experiments an experienced/trained subject was used to provide a qualitative analysis of the control method and highlight the main features. The method was validated on the elbow exoskeleton and the whole-arm exoskeleton systems. In the second set of experiments we used multiple healthy subjects to estimate its application in general use. These experiments were performed on the elbow exoskeleton system. The obtained experimental data was used to perform a statistical analysis regarding the method’s performance in terms of amplitude and frequency accuracy and adaptation time.

The feedback signal for updating each joint DoF trajectory was obtained according to Eqs (4)–(6) through the measurement of EMG signals of antagonist pairs of muscles in the corresponding joint. For the elbow exoskeleton we constructed the muscle activity feedback signal *U*(*t*) using the information of the human elbow joint behaviour. The antagonist pair of muscles that rotate the elbow joint and consequently forearm are *biceps brachii* and *triceps brachii*. Biceps muscles are performing flexion (i.e. decreasing the angle between arm and forearm), while triceps perform extension of the elbow joint (i.e. increasing the angle between arm and forearm). We used one electrode for each muscle group in the antagonist pair of muscles (two EMG channels altogether). We followed *SENIAM* [50] recommendations for the location of electrode placements. Biceps activation corresponded to positive change of elbow joint torque trajectory, while triceps corresponded to negative change.

In case of the whole-arm exoskeleton we used two active joints: elbow flexion/extension and shoulder flexion/extension. For elbow joint we used the same antagonist muscle pair as in case of elbow exoskeleton (two EMG channels). In case of shoulder joint control, we used the *deltoid* muscles that are located around the human shoulder joint. The anterior fibres of the deltoid are one of the primary flexors of the arm (i.e. lifting the arm). The activation signal from this muscle group corresponded to a positive change of trajectory. On the other hand, the posterior fibres of the deltoid are one of the primary extensors of the arm (i.e. lowering the arm). The activation signal from this muscle group corresponded to a change of trajectory in negative direction. We placed EMG electrodes to posterior and anterior deltoid (two EMG channels). Altogether, four EMG channels were used for experiments on whole-arm exoskeleton. Electrode placements were done in accordance with *SENIAM* recommendations [50].

Before the experiments we determined adaptation factor *G*_*u*_ experimentally and set it manually. We first empirically determined the suitable local value range of the *G*_*u*_ for the given hardware setup. We then defined several factors *G*_*u*_ within that value range. For each subject we performed preliminary trials using one of the predefined factors in each trial. Finally, we selected one of the predefined factors based on subject’s preference.

## Qualitative Validation Experiments

### Elbow Exoskeleton

**Fig 3:**
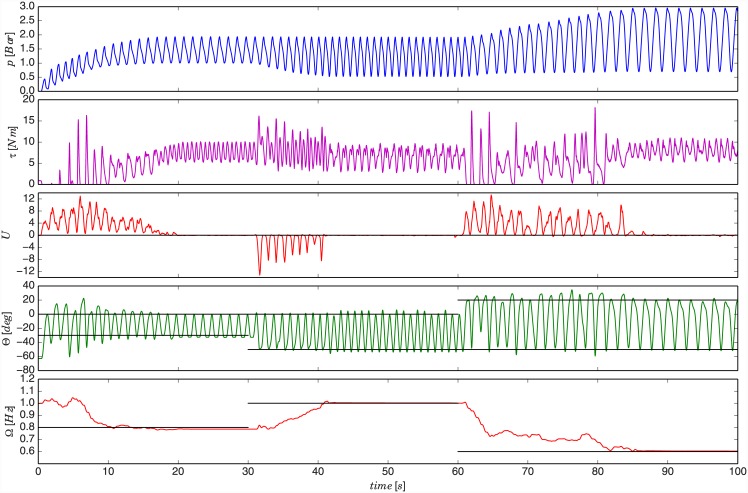
Results of experiment on elbow exoskeleton using a trained subject. The first graph shows commanded PAM pressure as it was learned by the DMP system. The second graph shows the joint torque calculated based on the force measurement from the load cell. The third graph shows the human muscle activity based feedback obtained from the measured EMG. The fourth graph shows the angle of the robot elbow joint as measured by the encoder in the exoskeleton joint. The fifth graph shows the frequency of motion as extracted by the adaptive oscillator from the muscle activity feedback *U*. The given referent states are highlighted by black lines.

**Fig 4:**
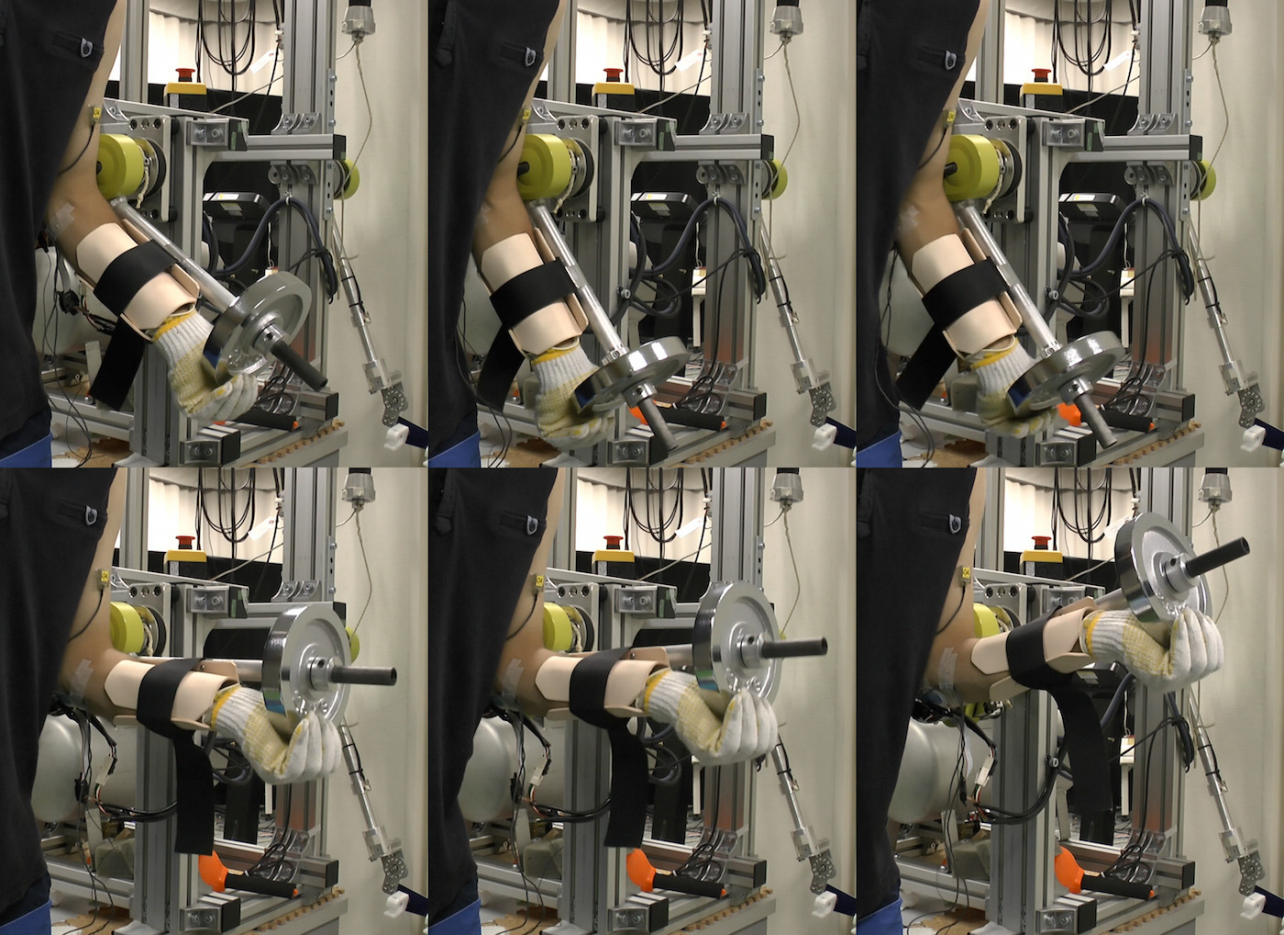
Sequence of photographs taken during the experiment that illustrate the state of the exoskeleton at the end of the 3 stages. Images in the first sub-row show the exoskeleton when it moved the object to the lower reference position, while the images in second sub-row show it in the upper position.

In the first experiment, the role of the exoskeleton was offloading the subject of muscular effort during the execution of the selected task. We used elbow exoskeleton to assist the trained subject in positioning of an object of an unknown mass using forearm motion. The object was a weight located at the subject’s hand. The task was to produce a reference motion defined at the beginning of the experiment. The experiment plan, given to the subject, was composed of three stages. In the first stage, the subject had to produce a motion amplitude in elbow joint between -30° and 0° at frequency of 0.8 Hz. Angle 0° corresponds to horizontal position of forearm while the arm is parallel to the body. In the second stage, the reference amplitude was between -50° and 0° and frequency 1 Hz. In the third stage, the reference amplitude was between -50° and 20° and frequency 0.6 Hz. The state of amplitude and frequency with respect to the reference was displayed to the subject in real-time on a monitor in front. For clarity, these reference states are highlighted by black lines in Fig 3. The state of the exoskeleton during the experiment at the end of each stage is shown by a sequence of photos in Fig 4. Please refer to S1 Video for a video of the experiment.

In the first stage, the subject had to start lifting the weight of the object to achieve the target angle motion trajectory (see Fig 3, forth graph). At the beginning PAM pressure control trajectory, as generated by the DMP system, was equal to zero throughout the entire phase (i.e. DMP weights in Eq (9) were equal to zero). The pressure trajectory is plotted in the first graph of Fig 3. While the subject was executing the task, the proposed control system gradually adapted the assistive behaviour of the exoskeleton to minimise the given muscle activity.

Initially, when the human started the effort of lifting the object the muscle activity feedback *U*(*t*) was high due to the contraction of the biceps muscle that flexed the elbow joint to overcome the gravitational force and other mechanical effects (see Fig 3, third graph). This feedback then updated the values of the weights according to Eq (15), which reshaped the trajectory to produce the assistive exoskeleton torque. When the human was lowering the object the muscle activity of the biceps muscle decreased as the motion was assisted by the gravity. Consequently, in this phase the DMP weights were updated considerably less as compared to the lifting phase. This procedure was periodically repeated while the trajectory gradually adapted to reduce the feedback signal.

The more assistive torque the exoskeleton produced, the less effort from the human muscles was required and consequently lower the feedback signal was. The adaptation speed can be controlled by the adaptation function *G*_*u*_ from Eq (6). For this experiment we set it experimentally to *G*_*u*_ = 60. Alternatively, the subject can potentially control the update speed indirectly by temporarily producing more/less effort than required for the given reference (i.e. temporarily offsetting the reference), if that is possible within the given task. When the robot provided full assistance (between 20 and 30 seconds), the feedback signal fell to zero and the trajectory stopped updating. At that moment the subject was instructed to relax and let the robot perform the learnt desired task until the reference changed in the transition to the second stage of the experiment.

We can confirm the described behaviour by observing the measured force from the load cell in-between PAM and the joint. We calculated the joint torque from the measured force and plotted it in the second graph of Fig 3. When the human lifted the object unassisted during the initial stage, the tendon between PAM and the joint was loosen and the force measured by the load cell was zero (i.e. torque was zero). The control algorithm then adapted to the muscle activity feedback by adding assistive power to the joint during the object-lifting phase. As PAM was gradually taking over the load, the tendon became stretched and the measured torque increased. Measured torque reached the peak at the transitions between the object-lowering and the object-lifting phase, as PAM had to compensate both gravitational force and inertial force associated with the transition. In some cases, there is a decrease of the measured torque as a result of non-ideal sine motion. For example, one such case was between 20 and 30 seconds of the experiment where the lower-end motion angle had a slightly flatter shape. This cased a momentary drop of angular acceleration, which caused a decrease of inertial torque and consequently a decrease of measured torque.

The task was defined only by reference amplitude and frequency, while the shape of the motion was not specifically prescribed. The subject successfully achieved the given task by producing bell-shaped motion, which is known to be characteristic (optimal) for human motion [51]. Thus, the learnt DMP successfully provided a reasonable torque to achieve the task motion.

The last graph of Fig 3 shows the frequency as estimated by the adaptive oscillator from EMG-based feedback. This frequency was used to control the execution frequency of the learnt trajectory. We can notice that the frequency of the oscillator remains at the last value if the muscle activity signal gradually falls to zero. We exploited this property of the adaptive oscillator to temporary keep the trajectory shape and make the exoskeleton reproduce the learnt behaviour when the human effort was fully compensated by the robot.

After the first stage, we demonstrated the adaptability of the proposed system by instructing the subject to change the existing trajectory. In the second stage, the subject had to expand the motion angle range in the negative direction to reach the new amplitude reference. In this case, the subject had to produce a negative muscle activity feedback. This was done by intuitively pressing the forearm against the exoskeleton support base to produce a motion corresponding to the new positional reference. The torque in the human elbow extension direction reflected in the activation of triceps muscle. The feedback signal then gradually reshaped the trajectory as seen in the time span between 30 and 40 seconds in the first graph of Fig 3. In addition, the subject had to increase the execution speed to reach the new target frequency.

**Fig 5:**
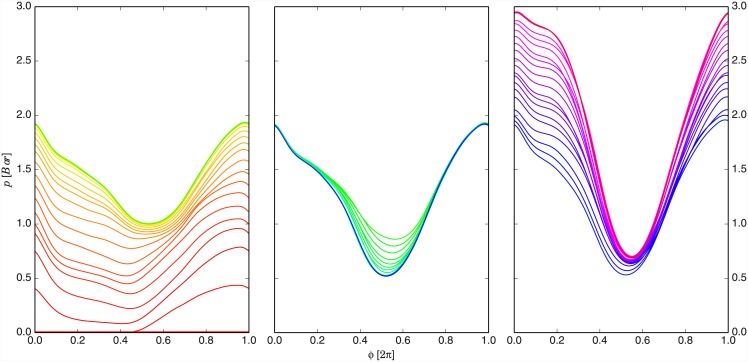
Control pressure trajectory updates as a function of phase. The graphs show trajectories as they were gradually learnt by the DMP system and fed to PAM pressure controller. Each colour variation represents one periodic cycle. The time sequence is illustrated by the colour spectrum, where the earlier trajectories are plotted with red and the later with violet colour. The left graph shows the first stage, the middle graph the second stage and the right graph shows the third stage of the experiment.

A similar task was then repeated in the third stage where the subject had to expand the motion trajectory in positive direction to achieve the new angle reference. In this case, the biceps muscle had to produce additional torque in the joint to increase the one that was already produced by the robot in the lifting phase. At the same time, the execution frequency had to be lowered to reach the new reference. This can be seen in Fig 3 between 60 and 85 seconds of experiment time. The evolution of the trajectory updates during all three stages of the experiment is shown in Fig 5, where the pressure trajectory is plotted as a function of phase.

### Whole-Arm Exoskeleton

With additional DoF, the control of exoskeleton joint motion must be coordinated properly to produce the desired arm end-effector motion. In cases of repetitive tasks, the frequencies of the joint motion trajectories must be in synchrony to produce the desired periodic motion of the end-effector. We used the robot’s shoulder and elbow joints to assist the corresponding human joints in the object-moving task where a periodic point-to-point end-effector motion was required. The object was a weight held in the human hand. The additional DoF allowed the human to arbitrary move the arm’s end-effector in sagittal plane within the kinematic constraints of human and robot arm and perform the given task. Please refer to S1 Video for a video of the experiment.

**Fig 6:**
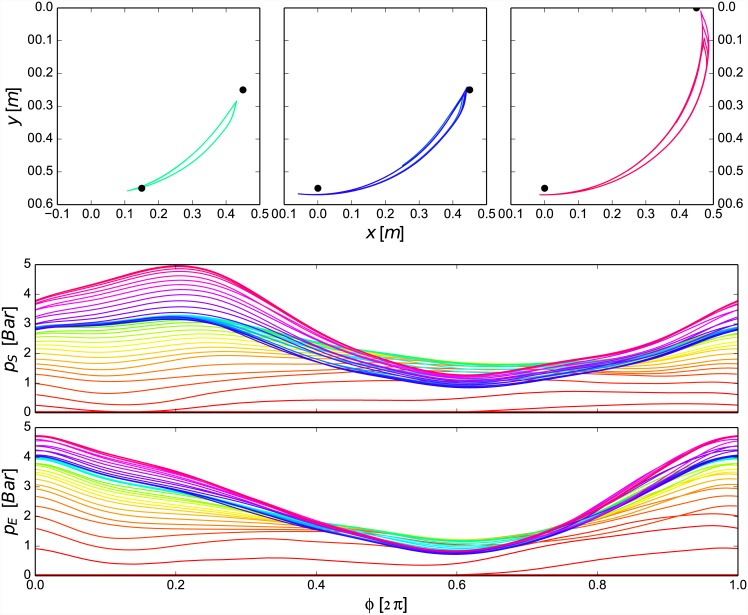
The three graphs in the first row show the end-effector motion at the end of there different stages of experiment (4 phases plotted for each). The red colour represents the earlier while violet represents the later trajectories. The lower two graphs show the control pressure trajectories updated as a function of phase for each joint.

**Fig 7:**
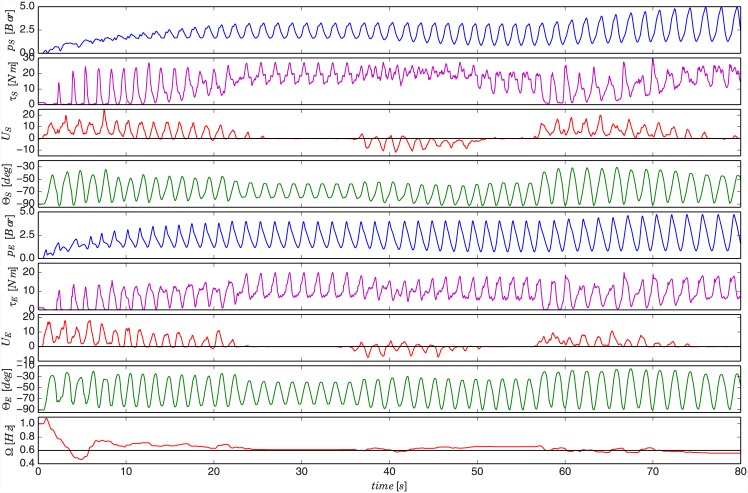
Results of experiment on whole-arm exoskeleton using a trained subject. The upper four graphs show the variables related to the shoulder joint. The lower five graphs show the variables related to the elbow joint. The last graph shows the estimated frequency from the elbow joint muscle activity feedback. Variable *p* corresponds to the commanded pressure learned by the DMP system, variable *τ* to the joint torques calculated from measured forces in the load cells, variables *U*_*S*_ and *U*_*E*_ to muscle activity feedback signals from shoulder and elbow flexion/extension joints, variable Θ to measured joint angles by the encoders and Ω to the frequency estimated by the adaptive oscillator from *U*_*E*_.

The human task in the experiment on the whole-arm exoskeleton was similar to the task in the first experiment on elbow exoskeleton. We divided experiment into three stages and selected several reference points in the sagittal plane that the subject had to reach with the end-effector (highlighted by the black points in Fig 6, second row graphs). In the first stage, the lower reference point of the end-effector was set to (0.15, -0.55) m with respect to the shoulder joint, while upper end-effector reference was set to (0.45, -0.25) m. In the second stage, the lower reference changed to (0, -0.55) m, while the upper remained the same. In the third stage, the lower reference remained as in the second stage, while the upper changed to (0.45, 0) m. The reference frequency of end-effector motion for the entire experiment was set to 0.6 Hz (highlighted with black line in Fig 7, last graph). The subject had a monitor in front to observe the actual states with respect to the reference conditions. The adaptation factor *G*_*u*_ was set experimentally to 40.

In the first stage of this experiment, the subject had to start moving the weight of the object to produce the end-effector trajectory that moved the object from one reference point to another. Initially, the muscular effort (i.e. anterior deltoid for the shoulder and biceps for the elbow joint) produced a high positive feedback signals *U*_*S*_ and *U*_*E*_, which shaped the respective assistive joint torque trajectories. In the second stage of the experiment, the subject was then instructed to reach the new reference point in the lower end of the end-effector trajectory. Consequently, the control method adapted the existing learnt trajectories to assist the subject in the new motion. In the third stage, the task was to reach the new reference point in the upper end of the trajectory. The angle readings in the fourth and eight graphs of Fig 7 confirm that the subject was able to change the motion in joint space.

**Fig 8:**
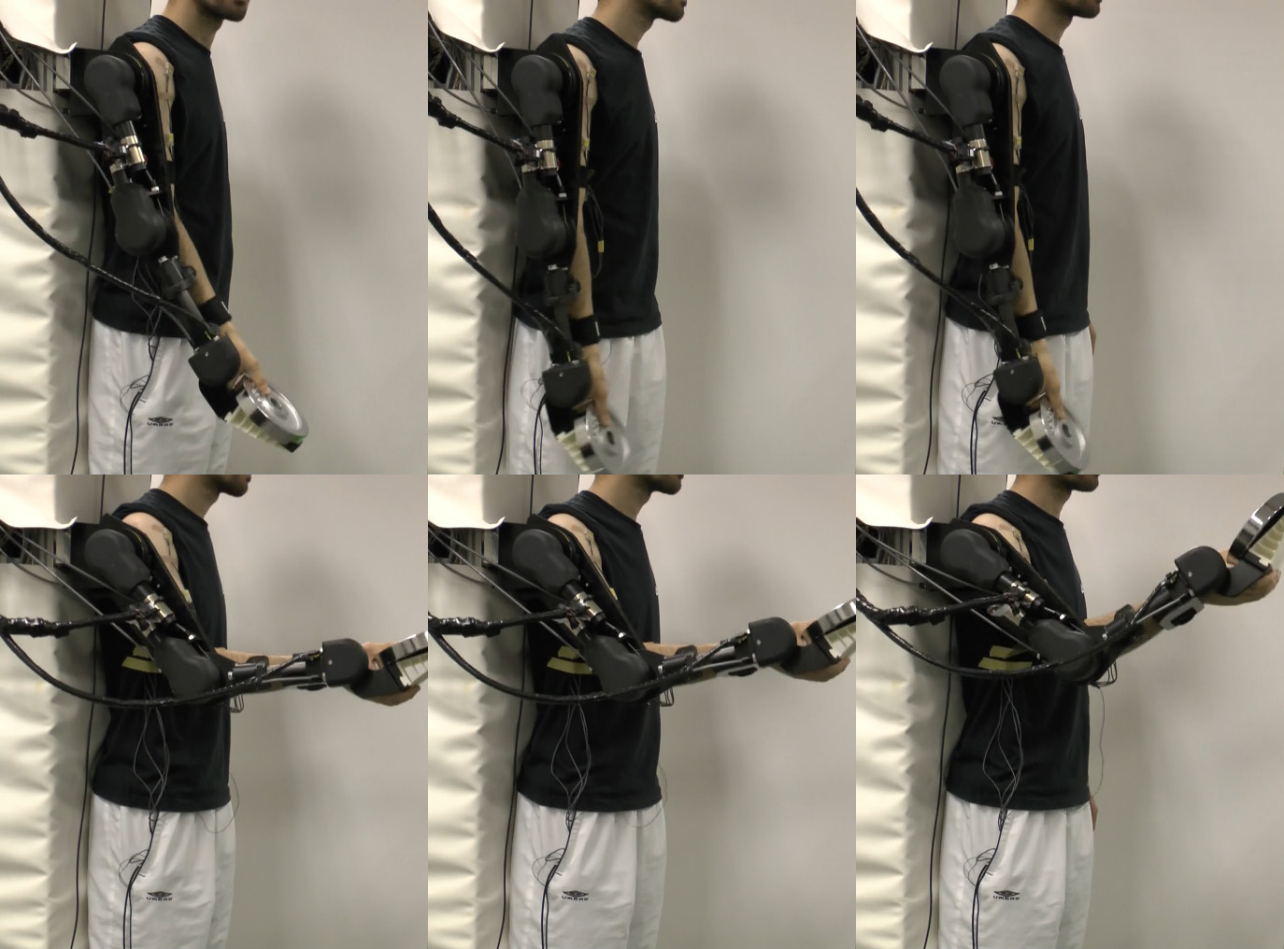
Sequence of photographs taken during the experiment that illustrate the state of the exoskeleton at the end of the 3 stages. Images in the first sub-row show the end-effector when it moved the object to the lower reference position, while the images in second sub-row show it in the upper position.

Fig 6 shows the results at different stages of the experiment. The graphs in the first row show the end-effector motion at the end of each stage. Here the left graph shows the motion of the end-effector at the end of the first stage of the experiment. In the middle graph there is the end-effector motion after the second stage. We can notice that the subject was able to adjust the motion of the end-effector in accordance to the changing reference points within a certain error range. This can also be confirmed by comparing the photographs taken during the experiment in Fig 8. The change of the pressure trajectories for each joint as a function of phase are shown in second and third row in Fig 6.

In the presented example, we used muscle activity feedback from a single joint (elbow) to extract the control phase and frequency for both joints. We controlled both joint trajectories with the extracted phase signal. With this we utilised the advantage of adaptive oscillators that allows synchronisation of multiple task-related signals with a single phase-estimation signal. Similarly, the human CNS can synchronise the different joint motion to produce a repetitive behaviour of upper limb end-effector [52]. We tested a possibility of extracting the phase and frequency for each joint individually. While the subject CNS was able to learn how to sufficiently maintain the synchrony between the two joint frequencies after several experiments, it imposes additional unnecessary effort on the exoskeleton operator.

## Multi-Subject Analysis

### Subjects and ethical statement

Eight healthy subjects participated in this study with an average age of 29.4 years (*SD* = 2.02 years), weight of 70.8 kg (*SD* = 2.01 kg) and height of 175.9 cm (*SD* = 1.29 cm). Prior to their participation, the subjects were informed about experimental procedures, potential risks and the aim of the study. Subjects signed an informed consent approved by Advanced Telecommunication Research Ethics Committee (Nos. 730, 731). The study was approved by Advanced Telecommunication Research Ethics Committee (Nos. 730, 731).

### Methods

The subjects were equipped with EMG electrodes as described in Overview of Experiments section. The task of the experiment was to move an object using forearm and elbow joint according to the predefined target amplitudes and frequencies. The first reference state was defined as moving the object between the angle of -40° and 0° at frequency of 1 Hz. The second reference state was defined as moving the object between the angle of -20° and 30° at frequency of 0.75 Hz. The reference and actual states were displayed to the subjects on a screen in real-time. To avoid any effects between the performances of the two reference states that could affect the comparison, we split the two reference states into two trials, with a fixed 10 minutes pause interval in-between. In particular, we wanted to avoid a potential fatigue, caused by the execution of the first reference state, affecting the performance of execution of the second reference state.

The experimental procedure was divided into two main modes: passive and active. In the passive mode each subject was instructed to move the object unassisted (i.e. the exoskeleton was passive) according to the two sets of reference states. In the active mode, the same was repeated while the proposed exoskeleton control method was employed to assist and then take over the motion. The subjects were first briefed about their experimental tasks. For each subject we first performed a minimum of three preliminary training trials in order for them to adapt to the exoskeleton system and control method. Adaptation factor *G*_*u*_ was set experimentally and tuned for each subject individually based on their preference.

In the analysis we compared the error between the given reference motion (i.e. amplitude and frequency) and the actual motion produced by each subject during the experiments. The amplitude error was defined as a deviation of the actual motion peaks from either the upper or lower reference. We normalised the amplitude error to the reference motion range. The frequency error was defined as a deviation of actual frequency of motion from the reference frequency for each sample time. We normalised frequency error to the given reference frequency.

In the passive mode we averaged the amplitude and frequency errors over five sequential periods of the experimental run. In the active mode, where the subjects controlled the exoskeleton using the proposed method, we averaged amplitude and frequency errors over five sequential periods after the muscle activity was minimised and the exoskeleton took over the repetitive execution of the task. In active mode we also analysed the adaptation time that was defined as a time from the beginning of the experiment to the moment where the subject’s muscles were relaxed and the exoskeleton took over the execution of the task.

The average values of the error parameters from each subject and each reference state were used in statistical analysis. A paired-samples t-test was used to determine whether there was a significant difference between the means of amplitude and frequency errors in passive and active mode. Differences between the errors were tested with post hoc t-tests with Bonferroni correction. The level of statistical significance was set to 0.05.

### Results

**Fig 9:**
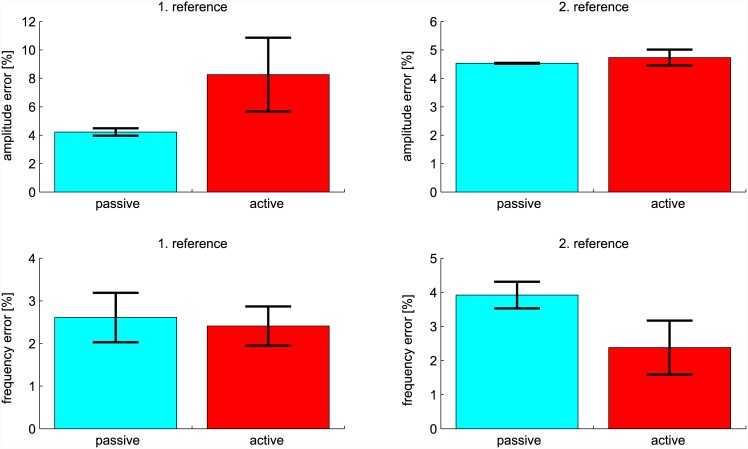
Results of the experiment on multiple subjects. Graphs are showing normalised tracking error of amplitude and frequency with respect to the given reference conditions in passive and active mode. Standard error of mean is marked with black.

The results of experiments are shown in Fig 9. We define statistical parameters as: *M* is mean, *SE* is standard error of mean; *t* and *p* are t-test parameters. On average, the amplitude error was larger in active mode (*M* = 8.26%, *SE* = 2.60%) compared to passive mode (*M* = 4.22%, *SE* = 0.26%) for the first reference state. The difference −4.03% was statistically not significant: *t*(7) = −2.32, *p* = .053. Comparing the amplitude error for passive (*M* = 4.53%, *SE* = 0.013%) and active mode (*M* = 4.73%, *SE* = 0.28%) for the second reference state, the difference −0.20% was statistically not significant: *t*(7) = −0.11, *p* = .91.

On average, the frequency was equally well maintained by the subjects in passive mode (1. reference (*M* = 2.61%, *SE* = 0.58%) and 2. reference (*M* = 3.92%, *SE* = 0.39%)) and by the proposed method controlling the exoskeleton in active mode (1. reference (*M* = 2.41%, *SE* = 0.46%) and 2. reference (*M* = 2.38%, *SE* = 0.79%)). The difference between passive and active mode for the first reference frequency 0.19% was not significant: *t*(7) = 0.18, *p* = .86. The difference for the second reference frequency 1.54% was not significant either: *t*(7) = 1.34, *p* = .22.

Mean adaptation time in the active mode was 31.0 s (*SE* = 3.70 s) for the first reference state and 31.3 s (*SE* = 3.29 s) for the second reference state. The adaptation time highly depends on the selected adaptation factor *G*_*u*_, which depended on the individual subject.

## Discussion

The results of the experiments show that the proposed control method can be successfully applied for solving the repetitive tasks. The users were able to utilise the control method to follow the given reference motion amplitude and frequency with accuracy comparable to the motion without the control method.

Results from multi-subject analysis indicate a larger amplitude error for the first reference state compared to the second reference state when using the proposed control method (see Fig 9 graphs in the first row). One of the possible causes for this may be that the first reference state experiments were performed first and consequently the subjects had less training compared to the second reference state experiment. This could suggest that a human sensorimotor learning was present during the experiments that allowed a co-adaptation between the human and the exoskeleton system. The subjects could improve their use of the proposed control method in terms of motion amplitude accuracy to a level comparable to the accuracy in passive mode.

The frequency error was roughly within the same range for passive and active mode. A slightly worse performance in terms of reference frequency tracking was observed in passive mode when subjects had to follow the second reference state (see Fig 9 graphs in the second row). This might potentially be attributed to a slightly more challenging motion range (between -20° and 30°) in the second reference state when subjects had to compensate the load themselves (i.e. passive mode). The load torque is the highest around 0° as the gravity vector is perpendicular to the lever (forearm). On other hand, the proposed method (active mode) can maintain a stable frequency when the appropriate behaviour is learnt.

The adaptive nature of the method allows the user to change the learnt feed-forward exoskeleton motion in real time according to the changing reference conditions. The adaptation rate of the exoskeleton is primarily defined by the adaptation factor *G*_*u*_ of Eq (6). High *G*_*u*_ allows larger updates and consequently faster adaptation rate. However, larger updates correspond to lower update resolution and may result in motion at lower accuracy. On the other hand, lower *G*_*u*_ yields higher update resolution and can be employed when more precise learning is required. However, this is at the expense of adaptation speed.

As of the current stage, the adaptation factor *G*_*u*_ of Eq (6) was determined experimentally and set manually. Future work will focus on developing more efficient methods to determine and optimise this parameter for a given hardware setup and conditions. In addition, a supplementary algorithm will be developed to enable automatic adjustment of this parameter based on other possible feedback modality. Particularly interesting direction would be to investigate the possibility of using biofeedback based on electroencephalography (EEG) as an additional command channel. This would give the user the ability to change the adaptation rate during the task execution depending on the preference.

Due to primarily feed-forward nature, the proposed method inherits some of the drawbacks related to feed-forward control, such as slow response to disturbances. While the method utilises feedback control to dynamically adapt the feed-forward control, the trajectory adaptation is gradual. For this reason the method is mainly suitable for tasks where gradual alterations are required. On the other hand, it may not be responsive enough to handle some emergency situations and external disturbances on its own. One such emergency situation is the case where an immediate stop at a certain point is required. This case can be solved by simply halting the control phase *ϕ* at that point [53]. A similar situation is the case of a sudden change of load (e.g. object held in hand is suddenly dropped). If the proposed control method was used in a regular mode, the user would have to exert an effort in the opposite direction of the previous load for some time in order to gradually readapt and reduce the learnt trajectory. In such a case, it may be more efficient to simply reset the trajectories by setting all DMP weights *w* to zero. Both situations can be potentially controlled by a higher-level controller or additional cognitive-level modality (e.g. EEG, voice command, etc.). Another interesting direction for solving unpredictable non-periodic situations would be to integrate methods that can recognise different action modes or motion patterns from the measured EMG signals [22, 23].

We shifted the frequency estimation from joint angle signal measured by the encoder to the muscle activity feedback in order to achieve better controllability. However, there is still a considerable coupling between the exoskeleton motion and muscle activity signal. Initially, some of the the naive subjects in this study experienced some difficulties controlling the target frequency using muscle activity feedback. Some training was required to become familiar with the control using the proposed frequency adaptation subsystem. A future research direction will focus on further improving the frequency estimation subsystem. The frequency can be also set by some external phase-control signal [53]. For example, phase could be controlled by a cognitive-level modality.

The method proposed in this paper mainly aims to solve and is best suited for exoskeleton control during the execution of repetitive tasks where occasional modifications of execution are required. This method could complement the control methods where muscle activity is used to directly control the exoskeleton motion [14, 15, 17, 19]. These effort-amplification methods are generally more responsive and provide a good control interface in cases of non-repetitive tasks, sudden unexpected events and emergency situations. However, they require continuous muscular effort even in case of repetitive tasks. On the other hand, our method aims to minimise the user’s effort once the assistive trajectories are learnt. In addition, the learnt trajectories can be stored for later reproduction. Compared to the control methods based on dynamical models [3, 5, 7, 11–13], the proposed method does not require complex models and adaptively derives the necessary feed-forward behaviour for specific tasks, hardware and conditions. A trade-off between advantages/disadvantages should be considered when selecting either method for specific tasks (refer to Table 1 for conceptual comparison between different types of control methods). To exploit the advantages of different methods, a higher-level controller would be required.

**Table 1:** Conceptual comparison between different types of control methods.

	responsive-ness	model complexity	continuous effort required	adaptability
**positive feedback**	high	low/high^1^	yes	high
**inverse dynamics**	low^2^	high	no^2^	low^3^
**proposed method**	low	low	no	high

^1^Depending on whether biomechanical or other models are required.

^2^Assuming that the desired motion is controlled by a feed-forward trajectory.

^3^In case the existing model is not sufficient for a new task or conditions.

In our analysis we selected a task of moving an object of unknown mass as it can be considered as a basic and fundamental element of manipulation. It can relate to common activity-of-daily-life tasks, such as object pick-and-place, table wiping, cutting vegetables, etc. While some of the tasks, such as pick-and-place task, may usually not require periodic behaviour, the task can still be learnt in a periodic manner and then the learnt trajectories can be repeated only once whenever the same task has to be executed. On the other hand, table wiping or cutting vegetables tasks are generally periodic with minor corrections. While in table wiping or cutting vegetables tasks, the gravity, object mass and changing arm configuration might not play as an important role as in pick-and-place tasks, the load is similarly unknown and changing as a result of interaction with the unstructured environment. Therefore, the adaptive nature of the proposed method should be well suited for solving such tasks.

## Conclusion

In the paper we proposed an approach for adaptive learning of exoskeleton joint torque behaviour. The approach uses EMG-based muscle activity feedback to include the human in the robot control loop. Muscle activity is not an estimation of an output torque but rather an information in which direction should the torque change to minimise the human muscular effort. This essentially makes the robot joint torque behaviour adapt to the current human behaviour. The proposed approach provides an interface between the human user and exoskeleton robot in body motion tasks. We showed its feasibility on two different mechanical systems designed for single and multiple DoF upper limb assistance.

The advantage of the proposed approach is that it can operate without predetermined behaviour, EMG-to-torque models or inverse dynamics models. Instead, it dynamically forms the joint torque trajectories to assist the operator in the desired task. If the task execution has to be altered, the method can adapt the robot behaviour to the new conditions without the need of breaking the operation. In case of using PAM actuators, the method can be used to bypass the need of pressure-to-torque models. However, if a suitable torque control is available or if different actuators are used, the method can be implemented to learn the torque directly.

## Supporting Information

S1 Video Video of qualitative validation experiments. The video shows the experiments on elbow exoskeleton and whole-arm exoskeleton using a trained subject as described in the paper.
(MP4)
